# Proactive and Reactive Recruitment of Black and Latino Adolescents in a Vaping Prevention Randomized Controlled Trial

**DOI:** 10.3390/children9070937

**Published:** 2022-06-22

**Authors:** Francisco Cartujano-Barrera, Ruthmarie Hernández-Torres, Rafael H. Orfin, Arlette Chávez-Iñiguez, Olga Alvarez Lopez, Chiamaka Azogini, Diana Bermudez, Evelyn Arana-Chicas, Xueya Cai, Scott McIntosh, Deborah J. Ossip, Ana Paula Cupertino

**Affiliations:** 1 Department of Public Health Sciences, University of Rochester Medical Center, Rochester, NY 14642, USA; Ruthmarie_Hernandez@urmc.rochester.edu (R.H.-T.); Rafael_Orfin@urmc.rochester.edu (R.H.O.); Arlette_Chavez@urmc.rochester.edu (A.C.-I.); Olga_AlvarezLopez@urmc.rochester.edu (O.A.L.); Chiamaka_Azogini@urmc.rochester.edu (C.A.); Evelyn_Arana@urmc.rochester.edu (E.A.-C.); Scott_McIntosh@urmc.rochester.edu (S.M.); Deborah_Ossip@urmc.rochester.edu (D.J.O.); Paula_Cupertino@urmc.rochester.edu (A.P.C.); 2 Hackensack High School, Hackensack Public Schools, Hackensack, NJ 07601, USA; DBermudez@hackensackschools.org; 3 Department of Biostatistics and Computational Biology, University of Rochester Medical Center, Rochester, NY 14642, USA; Xueya_Cai@urmc.rochester.edu

**Keywords:** adolescence, recruitment, tobacco control, vaping, Blacks, Latinos

## Abstract

The purpose of this study was to assesses the effectiveness of proactive and reactive methods in the recruitment of Black and Latino adolescents into a vaping-prevention randomized controlled trial (RCT). This study also assessed the characteristics of study participants by recruitment method. Proactive recruitment strategies included study presentations at community-based events (e.g., festivals, health fairs), school-based events (e.g., back-to-school events, after-school programs), and recreational centers (e.g., fitness centers, malls). Reactive recruitment strategies included study advertisements via social media (e.g., Facebook posts shared by local community-based organizations), word of mouth, and an academic-based research hub. Using proactive and reactive methods, in a 4-month period, 362 Black and Latino adolescents were successfully enrolled into the RCT. Compared to the proactive method, adolescents screened reactively were equally likely to be eligible but significantly more likely to enroll in the study. However, both proactive and reactive strategies made notable contributions to the overall recruitment effort. Moreover, proactive and reactive methods attracted adolescents with different characteristics (e.g., age, gender, sexual orientation, etc.). These findings suggest that both proactive and reactive recruitment strategies should be implemented for studies interested in recruiting a diverse sample of Black and Latino adolescents.

## 1. Introduction

Adolescence (ages 10 to 19 years) [1] is a period of life characterized by tobacco- and nicotine-use initiation, experimentation, and progression to long-term addicted use [2,3,4,5]. According to the United States (U.S.) National Youth Tobacco Survey, in 2018, 7.2% of middle school students (approximately 840,000) and 27.1% of high school students (approximately 4 million) reported current use of any tobacco product and electronic nicotine delivery systems (ENDS) [6]. Electronic cigarettes (e-cigarettes) were the most used tobacco/nicotine products among middle school (4.9%) and high school (20.8%) students [6]. There is sufficient evidence that e-cigarette use (vaping) during adolescence is associated with future initiation of cigarette, marijuana, and alcohol use [7,8,9]. Moreover, early nicotine exposure puts adolescents at risk for a lifetime of vaping addiction as well as unknown health risks of long-term e-cigarette use. Chemical and heavy metal exposure from e-cigarettes and risk of toxicity and acute injuries are a public health concern [10,11,12,13,14,15]. 

Despite the potential adverse effects and high prevalence of e-cigarette use among adolescents, there is limited research on effective strategies in the scope of prevention. Moreover, the limited research on vaping prevention is hampered by methodological limitations, such as the underrepresentation of Black and/or Latino individuals, a lack of multi-modal recruitment strategies, and scarce reporting of recruitment data. Examples from the available literature include one study assessing different message themes to prevent vaping among adolescents and young adults [16]. Recruitment was solely conducted via Qualtrics Online Sample, a commercial survey sampling and administration company [17]. This effort resulted in the recruitment of 1564 adolescents (20.5% self-identified as Hispanic/Latino, and 13.4% self-identified as African American/Black) [16]. A second example is a study evaluating a school-based vaping educational program delivered by health professionals with vast experience in conducting tobacco education [18]. Participants were exclusively recruited from a mix of public and private schools in urban and rural settings in Alabama [18]. This effort resulted in the recruitment of 2889 middle and high school students (no reported data on race nor ethnicity) [18]. A third example is a study developing and pilot testing a risk communication campaign to prevent vaping among adolescents [19]. Participants were recruited through direct email invitations from the regional YMCA partner network and electronically distributed invitations via social media channels proctored by local teen-serving, community-based organizational networks [19]. The final sample consisted of 268 participants (9.3% self-identified as Hispanic, and 26.1% self-identified as Black/African American) [19]. While these three studies have been an extraordinary addition to the expanding literature on vaping prevention, increasing study participation of Black and Latino individuals, diversifying recruitment strategies, and detailing recruitment data are necessary to achieve research generalizability, facilitate evidence-based policy, improve health outcomes, and advance health equity.

Literature on the recruitment of Black and Latino individuals into tobacco-control (e.g., smoking cessation) studies have described the use of different recruitment methods [20,21,22,23,24,25,26]. These studies have recruited Black and Latino participants through proactive (also known as active or direct) recruitment, which includes direct interaction with potential participants [27], and reactive (also known as passive or indirect) recruitment, where individuals contact the study themselves [24]. Proactive strategies often include telephone calls, staff attendance at local health fairs, and local community presentations [27]. Reactive strategies often involve the distribution of printed material, such as flyers, newspaper ads, and advertisements in church bulletins, and use of media outlets, such as radio and television [24]. Proactive recruitment usually requires more resources but may yield higher rates of accrual, whereas reactive methods may be less effortful but result in lower rates of accrual [28]. It is also possible that the different recruitment approaches will yield participants with different characteristics. To the best of our knowledge, no study has assessed the effectiveness of proactive and reactive strategies in the recruitment of Black and Latino adolescents into vaping-prevention studies. The present study assesses the effectiveness of proactive and reactive methods and strategies in the recruitment of Black and Latino adolescents into a vaping-prevention randomized controlled trial. In this study, recruitment method refers broadly to either proactive or reactive recruitment. Recruitment strategies refer to the specific recruitment type implemented within each recruitment method. This study also assesses the characteristics of study participants by recruitment method.

## 2. Materials and Methods

### 2.1. Study Design

We conducted a randomized controlled trial (RCT) to assess the impact of theoretical-based graphic messages at preventing vaping among 362 Black and Latino adolescents. The graphic messages were developed using participatory research methods and incorporate four main theoretical constructs: health reward, financial reward, self-efficacy, and social norms [29]. Participants were compensated with a USD 25 gift card for their time and effort. This manuscript compares proactive and reactive recruitment methods for participants enrolled in the RCT. Study procedures were approved and monitored by the University of Rochester Medical Center Institutional Review Board (STUDY00006267). The trial was registered on ClincialTrials.gov (NCT04899999).

### 2.2. Recruitment

Recruitment was conducted by a team of diverse (e.g., race, ethnicity, and gender), bilingual (English and Spanish), trained recruiters. Recruitment started in August 2021 and ended in December 2021. Proactive recruitment strategies included study presentations supported by a display table at community-based events (e.g., festivals, health fairs), school-based events (e.g., back-to-school events, after-school programs), and recreational centers (e.g., fitness centers, malls). At the end of each study presentation, recruiters asked individuals if they were interested in participating in the RCT. The proactive recruitment strategies mostly took place in Hackensack, New Jersey, and Rochester, New York. U.S. Census Bureau data for the city of Hackensack, New Jersey, indicate lower proportions of White (49.7% vs. 72.1%) and more African American/Black (24.1% vs. 15.0%) as well as Hispanic/Latino (36.3% vs. 20.4%) residents than statewide, more residents speaking languages other than English at home (48.6% vs. 31.0%), and those living in poverty (12.3% vs. 10.0%) [30]. U.S. Census Bureau data for Rochester, New York, indicate lower proportions of White (45.4% vs. 69.6%) and more African American/Black (39.4% vs. 17.6%) residents than statewide. The proportion of Hispanic/Latino residents is similar in Rochester and New York (19.4% and 19.3%, respectively). Compared to New York State, more residents of Rochester live in poverty (30.4 vs. 12.7%) [31]. One school-based event occurred in Ponce, Puerto Rico, where almost all students were Hispanics/Latinos.

Reactive recruitment strategies included study advertisements via social media (e.g., Facebook posts shared by local community-based organizations), word of mouth, and an academic-based research hub (e.g., UR Health Research—An institutional resource to promote participation in clinical trials). UR Health Research connects individuals to clinical trials, promotes research findings, and educates the community about health research. No paid online advertisements via social media (e.g., Facebook ads) were used.

### 2.3. Parents’/Guardians’ Permission 

Individuals who were interested in participating in the study received two informational letters, namely one addressed to the adolescent and the other to the parents/caregivers. The informational letters (available in Spanish and English) described the study, explained the benefits and risks, and included the study team contact information. Once the parents/caregivers received and reviewed the informational letter, they were instructed to contact the study team via electronic email or phone call. Research staff then scheduled a phone call with interested parents/caregivers to obtain permission for their adolescent to participate. During the phone call, the research team discussed all aspects of study participation and confidentiality and answered any questions. Parents/caregivers and adolescents were informed that they were free to decline study participation and end their involvement at any time without negative consequences. Once the parents/caregivers gave verbal permission for their adolescent to participate, study staff contacted the adolescent to conduct the eligibility assessment. 

### 2.4. Eligibility

Individuals were eligible if they (1) self-identified as African American/Black and/or Hispanic/Latino, (2) knew how to read and speak English and/or Spanish, (3) were at least 12 but no greater than 17 years old, (4) had never used e-cigarettes, and (5) had access to a device that would allow them to connect to the online survey (e.g., desktop, laptop, tablet, and/or smartphone). Exclusion criteria included (1) not identifying as African American/Black and/or Hispanic/Latino and (2) currently using e-cigarettes. Eligibility assessment was conducted by study staff in the adolescent’s language of preference: either English or Spanish.

### 2.5. Participants’ Assent

Adolescents who were eligible to participate in the study received a unique link to REDCap, a secure web application for building and managing online surveys and databases [32]. Through this link, the adolescents accessed the informational letter described above (available in English and Spanish). Adolescents assented to participate in the study by clicking “Yes, I assent to participate in the study” via REDCap after reading the informational sheet. In this study, enrolled participants refer to adolescents who assented to participate in the study.

### 2.6. Assessments

All assessments were completed in the participants’ language of preference, either English or Spanish. Assessments were adapted from surveys used in previous studies and pre-tested for survey administration among the study team [33]. The baseline survey collected information on demographics (e.g., race, ethnicity, age, gender, sexual orientation, state of residence, and employment status). Participants’ state of residence was grouped into one of five regions (e.g., Northeast, Midwest, South, West, and Puerto Rico), in accordance with the U.S. Census Bureau [34]. Informed by prior foundational research on youth electronic cigarette use, susceptibility to future vaping was assessed with three items tapping curiosity, intent, and social influence [33]. Participants were asked: “Have you ever been curious about using e-cigarettes/vaping?”; “Do you think that you will use e-cigarettes/vape in the next 12 months?”; and “If one of your best friends were to offer you an e-cigarette/electronic vapor product, would you use it?” (1 = “Definitely not” to 4 = “Definitely yes”). These response categories were combined to create a dichotomous variable on susceptibility to future vaping (1 = “Definitely not”; 2 = “Probably not”, “Probably yes”, and “Definitely yes”). Moreover, we merged these items to create an overall susceptibility variable. Participants who responded with a response other than “Definitely not” to one or more items were deemed susceptible to future vaping.

### 2.7. Analyses

For each recruitment method, characteristics of enrolled participants were summarized with percentages for categorical variables and with means and standard deviations (SD) for continuous variables. Rates of eligibility and enrollment across the two recruitment methods were compared using chi-square tests. Differences in categorical variables were exploratorily compared using Pearson’s chi-square tests or Fisher exact test, while differences in continuous variables were compared using one-way ANOVA tests or Nonparametric Wilcoxon test depending on data distributions. Logistic regressions were used to estimate relative risks and 95% confidence intervals to measure the (1) association between recruitment method and the likelihood of obtaining eligible individuals among screened individuals and the (2) association between recruitment method and the likelihood of enrolling the screened participants. Intention-to-treat (ITT) was applied for missing data, and all analyses were performed in SPSS 14.0.

## 3. Results

Of the 408 individuals who completed screening, 401 (98.2%) met eligibility criteria, and 362 (88.7%) enrolled in the RCT. Most participants were enrolled via reactive recruitment strategies (*n* = 204, 56.4%) compared to the proactive recruitment strategies (*n* = 158, 43.6%). Table 1 lists the numbers of individuals who were screened and enrolled by recruitment method and includes measures (ratios) of eligibility efficiency and enrollment efficiency. Compared to the proactive method, individuals screened in the reactive method were equally likely to be eligible (98.1% vs. 98.4%; RR = 0.81, 95% CI = 0.18–3.58, *p* = 0.29) but statistically significantly more likely to enroll in the study (96.2% vs. 80.6%; RR = 5.13, 95% CI = 2.46–10.74, *p* < 0.001).

Table 2 shows the efficiency of specific strategies used in both recruitment methods. In both proactive and reactive methods, all strategies yielded extremely high eligibility efficiency ratios (97.5–100%) except for academic-based hub (42.8%). Within the proactive method, the strategy that yielded the highest enrollment efficiency ratio was school-based events (86.9%). Within the reactive method, social media and word of mouth yielded extremely high enrollment efficiency ratios (100% and 97.0%, respectively). 

At baseline, participants’ mean age was 14.9 years old (SD 1.4); 50% of participants self-identified as African American/Black, and 50% self-identified as Hispanic/Latino (Table 3). Almost two-thirds of participants (64.9%) were male, 87.5% were heterosexual, and 66.3% lived in the Northeast region. Most participants (82%) selected English as their language of preference, and 18.7% were currently employed. More than half of participants (54.7%) were classified as susceptible to future vaping.

Table 3 compares the characteristics of enrolled participants between both recruitment methods. Black participants were significantly more likely to be recruited reactively compared to their non-Black counterparts (*p* < 0.001). Latino participants were significantly more likely to be recruited proactively compared to their non-Latino counterparts (*p* < 0.001). Participants recruited reactively were older (*p* < 0.001), more likely to be male (*p* < 0.001), more likely to be heterosexual, more likely to select English as their language of preference (*p* < 0.001), more likely to be currently employed (*p* = 0.019), and more likely to be susceptible to future vaping (*p* = 0.027) compared to participants recruited proactively.

## 4. Discussion

In a 4-month period, 362 Black and Latino adolescents were successfully enrolled into an RCT testing the impact of theoretical-based graphic messages at preventing future vaping. To the best of our knowledge, this is the first study to describe the effectiveness of diverse strategies for identifying and recruiting Black and Latino adolescents into a vaping-prevention RCT. This study compared proactive and reactive recruitment strategies on eligibility and enrollment rates. Results of this study show that compared to the proactive method, adolescents screened reactively were equally likely to be eligible but significantly more likely to enroll in the study. However, both proactive and reactive strategies made notable contributions to the overall recruitment effort.

The overall greater enrollment efficiency of reactive strategies compared to proactive strategies is consistent with other tobacco-control research among Black and Latino adults [23,24,35,36]. As noted by Harris et al., reactive recruitment may be more effective among underrepresented minorities due to its nature of reaching a wider audience and individuals’ readiness and motivation to participate in tobacco-control studies [24]. Of the different recruitment strategies, all strategies in both proactive and reactive methods yielded extremely high eligibility efficiency ratios (97.5–100%) except for academic-based hub (42.8%). In order to reach the recruitment goal, study staff attended an array of events and sites that were known to garner the attendance of Black and Latino adolescents. Proactive strategies (e.g., attending community-based events, school-based events, and recreational centers) resulted in recruitment of 43.6% of the sample. Within the proactive method, the strategy that yielded the highest enrollment efficiency ratio was school-based events (86.9%). As described by Tingen et al., schools—especially elementary and middle schools—provide a promising setting for reaching adolescents and parents/caregivers for tobacco-control studies [37]. In contrast, reactive strategies resulted in 56.4% of the sample. Within the reactive method, word of mouth and social media yielded extremely high enrollment efficiency ratios (97% and 100%, respectively). As described by Bonevski et al., Black and Latino individuals who do not receive care, information, or support from community-based organizations (CBOs) or academic medical centers may be less exposed to research opportunities, and thus, they might be less likely to participate in research [38]. Therefore, word of mouth may be uniquely fitted to enhance research engagement among Black and Latino individuals who have lower exposure to research. Furthermore, the extremely high efficiency of social media—specifically Facebook—is no surprise, as a systematic review of Facebook as a recruitment tool for adolescent health research showed that it is a successful method [39]. Moreover, social media is an integral part of adolescents’ lives, as 82% of them use social media to engage in fundamental tasks (e.g., learning), identity formation, and social connectivity (e.g., engaging with friends [40]). It is important to acknowledge that in this study, recruitment via social media only occurred through Facebook. Future studies should assess the impact of recruiting Black and Latino adolescents via Instagram, Snapchat, and TikTok—the currently most-used social media platforms among adolescents [41].

In this study, proactive and reactive methods attracted adolescents with different characteristics. Black adolescents were significantly more likely to be recruited reactively compared to proactively. This result is consistent with previous studies indicating that, among Black adults, reactive recruitment is the most successful recruitment method in tobacco-control (e.g., smoking cessation) studies [26,35,36]. In contrast, Latino adolescents were significantly more likely to be recruited proactively. Surprisingly, this result is inconsistent with previous studies showing that, among Latino adults, reactive strategies are the most successful recruitment efforts in tobacco-control (e.g., smoking cessation) studies [23,36]. However, García et al. reviewed the recruitment strategies of five studies with Latinos related to type 2 diabetes and tuberculosis and found that the recruitment efforts were far more successful when they relied on methods of direct contact with potential participants [42]. This was because Latino participants and recruiters shared cultural values of *personalismo* (preference for warm relationships that convey care and acceptance of participants and their circumstances), *simpatía* (preference for smooth relationships that are free of confrontation and criticism as well as finding someone likeable, sharing common interests), *respeto* (respect), *confianza* (trust), and *familismo* (emphasis on family, cooperation, loyalty, and interdependence) [42,43]. 

In this study, female participants were significantly more likely to be recruited via proactive strategies compared to reactive strategies. This result is consistent with other studies that have found that proactive approaches are successful to recruiting female adolescents from minority backgrounds [44,45]. Moreover, in this study, participants recruited reactively were more likely to be heterosexual compared to participants recruited proactively. The National Institutes of Health has recognized sexual and gender minority people as a “health disparity population for research [46]” because they experience numerous health and health care inequities, including worse mental health outcomes [47,48,49,50] and low utilization of preventive care services [51,52,53]. Future studies aiming to recruit adolescents from diverse sexual orientations (e.g., lesbian, gay, bisexual) can build upon proactive approaches to conduct tobacco-control research among non-heterosexual adolescents. Additionally, participants recruited reactively were significantly older, more likely to select English as their language of preference, and more likely to be currently employed compared to participants recruited proactively. The statistical difference observed in the region could be due to the proactive recruitment strategies occurring in the Northeast region (e.g., New Jersey and New York), with the exception of a school-based event in Puerto Rico that recruited 13 adolescents. Lastly, participants’ overall susceptibility to future vaping is consistent with the data reported among adolescents attending public schools in a low-income community in New Jersey [33].

The study team included Black and Latino researchers and staff. Thus, factors that contributed to recruitment success may include race, ethnicity, and language matching of research staff to both parents/caregivers and adolescents. In addition, recruitment efforts involved close partnership with key leaders of the Black and Latino communities, and study materials were culturally relevant and language appropriate. As recommended by Rhodes et al., emphasizing the study uniqueness was a vital recruitment tactic [54]. Recruiters in this study stressed to parents/caregivers and adolescents that the study was specifically designed and developed for Black and Latino adolescents and that no other vaping-prevention study like it existed for these populations. Additionally, key to recruitment was the provision of monetary compensations to study participants. As described by Ozer, monetary compensation increases youth’s feelings of equity, value, and investments in research projects [55].

### 4.1. Study Relevance and Implications for Future Research

This study adds valuable insights to the expanding literature on vaping prevention. Study results demonstrate that it is feasible to recruit Black and Latino adolescents—two traditionally hard-to-reach groups—into a vaping-prevention RCT. These findings provide evidence of Black and Latino adolescents being interested in participating in vaping-prevention research if recruited in a culturally and linguistically appropriate manner. Proactive and reactive methods attracted adolescents with different characteristics (e.g., age, gender, sexual orientation, etc.). An appreciation of these differences is important, as this information could be utilized to tailor recruitment strategies for Black and Latino adolescents. This study is timely given that Black and Latino adolescents are often underrepresented in tobacco-control studies, and this study suggests that 55% of them are susceptible to future vaping. Moreover, Black and Latino communities have a history of being targeted by the tobacco industry [56,57]. For example, vape shops are more densely distributed in neighborhoods with large Black and Latino populations [58,59]. Lastly, these recruitment approaches among Black and Latino adolescents have the potential to be replicated for research involving other public health concerns.

### 4.2. Strengths and Limitations

To the best of our knowledge, this is the first study to purposely focus on African American/Black and Hispanic/Latino adolescents for a vaping-prevention RCT. It is important to acknowledge that in the U.S., African American/Black is typically assessed as a race and Hispanic/Latino as an ethnicity and that these groups are not mutually exclusive. In this study, we did not encounter participants who self-identified as both African American/Black and Hispanic/Latino.

One of the strengths of this study and a possible facilitator of recruitment is that this research builds upon our established history of smoking-cessation treatment and research within the community [20,60,61,62,63,64]. Moreover, this work is grounded in principles of community-based participatory research (CPBR). CBPR is a partnership approach to research that involves community members, organizational representatives, and researchers across all phases of research [65]. This approach ensures that the research is relevant, meaningful, and appropriate to the population for which it is planned [65]. Consistent with CBPR, we recognize the community as a unit of identity and build on its unique strengths and resources. Specifically, the community (1) has identified vaping as a health concern, (2) is engaged in intervention design, (3) guides the research team to effective recruitment strategies, (4) collaborates in interpreting findings, and (5) cooperates in disseminating findings. 

Another strength of the study is the use of proactive and reactive methods for recruitment. The design of the study allowed us to isolate and determine the unique contribution of each recruitment approach while tracking critical denominators to calculate both eligibility and enrollment efficiency. The inclusion of Spanish-speaking participants is another study strength: 18% of participants in this study selected Spanish as their language of preference. Despite these strengths, the current study has several methodological limitations. First, this study was not designed to test the efficiency of recruitment strategies. Given the broad reach of our channels, it is possible that participants were exposed to multiple strategies. It is also possible that individuals responding reactively may have been exposed to a prior or concomitant proactive strategy. Therefore, some cross-contamination effect cannot be ruled out. Lastly, we did not conduct a cost effectiveness analysis in this study because we did not track total costs and time spent. Moreover, the study relied on volunteers and the support of CBOs. We acknowledge that an essential component in determining the effectiveness of one recruitment method over another is to consider the costs and time spent. For example, the high eligibility and enrollment efficiency ratios of social media in this study are very promising given that no paid online advertisements (e.g., Facebook ads) were used. Moreover, the study flyer that was shared on social mediums was designed by the research staff and not a professional graphic designer. In contrast, all proactive recruitment strategies demanded staff resources, and results were mixed. For example, one recruiter (R.H.O.) attended one event at the Boys and Girls Club of Rochester—a local recreational center—for one hour. At this event, the recruiter identified 12 individuals who were interested in participating in the RCT. All 12 individuals were eligible to participate in the RCT and enrolled in the study; the eligibility and enrollment efficiency ratios of this particular event were both 100%. Another example was a back-to-school event organized by the Eugenio María de Hostos Charter School. Two recruiters (R.H.-T. and A.P.C.) attended the 4-hour event and identified 22 individuals who were interested and eligible to participate in the RCT (eligibility efficiency ratio of 100%). Of the 22 individuals, 13 were enrolled in the study (enrollment efficiency ratio was 59%). A third example was a community-based event at the International Plaza of the City of Rochester to celebrate Hispanic Heritage Month. Two recruiters (R.H.O. and A.C.-I.) attended the 3-hour event and did not identify a single individual who was interested in participating in the study.

## 5. Conclusions

Using proactive and reactive methods, a heterogeneous sample of Black and Latino adolescents was enrolled in a vaping-prevention RCT. Compared to the proactive method, adolescents screened reactively were equally likely to be eligible but significantly more likely to enroll in the study. However, both proactive and reactive strategies made notable contributions to the overall recruitment effort. Moreover, proactive and reactive methods attracted adolescents with different characteristics (e.g., age, gender, sexual orientation, etc.). These findings suggest that both proactive and reactive recruitment strategies should be implemented for studies interested in recruiting a diverse sample of Black and Latino adolescents.

## Figures and Tables

**Table 1:** Efficiency ratios for proactive and reactive recruitment methods.

Recruitment Method	Number Screened	Number Eligible	Number Enrolled	Eligibility Efficiency Ratio ^a^	Enrollment Efficiency Ratio ^b^
Proactive	196	193	158	98.4	80.6
Reactive	212	208	204	98.1	96.2
Total	408	401	362	98.2	88.7

^a^ Ratio of number eligible to number screened. ^b^ Ratio of number enrolled to number screened.

**Table 2:** Recruitment efficiency of specific recruitment strategies.

Recruitment Method	Recruitment Strategy	Number Screened	Number Eligible	Number Enrolled	Eligibility Efficiency Ratio ^a^	Enrollment Efficiency Ratio ^b^
Proactive	Community-based events	31	31	23	100	74.1
	School-based events	123	120	107	97.5	86.9
	Recreational centers	42	42	28	100	66.6
Reactive	Social media	71	71	71	100	100
	Word of mouth	134	134	130	100	97.0
	Academic-based hub	7	3	3	42.8	42.8

^a^ Ratio of number eligible to number screened. ^b^ Ratio of number enrolled to number screened.

**Table 3:** Baseline characteristics of enrolled participants who were recruited using proactive and reactive strategies.

Characteristic	Total (*N* = 362)	Proactive (*n* = 158)	Reactive (*n* = 204)	*p*-Value
	Mean (SD)	Mean (SD)	Mean (SD)	
Age	14.9 (1.4)	14.6 (1.55)	15.2 (1.37)	<0.001
	***N* (%)**	***n* (%)**	***n* (%)**	
*Race*: African American/Black	161 (50%)	42 (26.6%)	139 (68.1%)	<0.001
*Ethnicity*: Hispanic/Latino	161 (50%)	116 (73.4%)	65 (31.9%)	<0.001
*Gender:* Male	235 (64.9%)	77 (48.7%)	158 (77.5%)	<0.001
*Sexual orientation:* Heterosexual	317 (87.5%)	125 (79.1%)	192 (94.1%)	<0.001
*Region*: Northeast	240 (66.3%)	145 (91.8%)	95 (46.6%)	<0.001
*Language of preference*: English	297 (82%)	101 (63.9%)	196 (96.1%)	<0.001
Currently employed	68 (18.7%)	21 (13.3%)	47 (23.0%)	0.019
Susceptible to future vaping	198 (54.7%)	76 (48.1%)	122 (59.8%)	0.027

## Data Availability

The datasets generated for this study are available on request to the corresponding author.
